# Care of patients undergoing withdrawal of life-sustaining treatments: an ICU nurse perspective

**DOI:** 10.1186/s12912-024-01801-7

**Published:** 2024-03-04

**Authors:** Sung Ok Chang, Dayeong Kim, Yoon Sung Cho, Younjae Oh

**Affiliations:** 1 College of Nursing and BK21 FOUR R&E Center for Learning Health Systems, Korea University, Seoul, Republic of Korea; 2 Korea University Guro Hospital, Seoul, Republic of Korea; 3 College of Nursing and Research Institute of Nursing Science, Hallym University, Hallymdaehakgil 1, 24252 Gangwon-do, Republic of Korea

**Keywords:** Life-sustaining treatment, Nurse, ICU, Discourse analysis

## Abstract

**Background:**

Intensive care unit (ICU) nurses working in South Korea report experiencing uncertainty about how to care for patients undergoing withdrawal of life-sustaining treatments (WLT). A lack of consensus on care guidelines for patients with WLT contributes to uncertainty, ambiguity, and confusion on how to act appropriately within current law and social and ethical norms. To date, little has been discussed or described about how ICU nurses construct meaning about their roles in caring for dying patients in the context of wider social issues about end-of-life care and how this meaning interacts with the ICU system structure and national law. We aimed to better understand how ICU nurses view themselves professionally and how their perceived roles are enabled and/or limited by the current healthcare system in South Korea and by social and ethical norms.

**Methods:**

This qualitative descriptive study was conducted using in-depth, semi-structured interviews and discourse analysis using Gee’s Tools of Inquiry. Purposive sampling was used to recruit ICU nurses (*n* = 20) who could provide the most insightful information on caring for patients undergoing WLT in the ICU. The interviews were conducted between December 2021 and February 2022 in three university hospitals in South Korea.

**Results:**

We identified four categories of discourses: (1) both “left hanging" or feeling abandoned ICU nurses and patients undergoing WLT; (2) socially underdeveloped conversations about death and dying management; (3) attitudes of legal guardians and physicians toward the dying process of patients with WLT; and (4) provision of end-of-life care according to individual nurses’ beliefs in their nursing values.

**Conclusion:**

ICU nurses reported having feelings of ambiguity and confusion about their professional roles and identities in caring for dying patients undergoing WLT. This uncertainty may limit their positive contributions to a dignified dying process. We suggest that one way to move forward is for ICU administrators and physicians to respond more sensitively to ICU nurses’ discourses. Additionally, social policy and healthcare system leaders should focus on issues that enable and limit the dignified end-of-life processes of patients undergoing WLT. Doing so may improve nurses’ understanding of their professional roles and identities as caretakers for dying patients.

**Supplementary Information:**

The online version contains supplementary material available at 10.1186/s12912-024-01801-7.

## Introduction

The landscape of discourse about death and dying in South Korea and other countries has changed dramatically in the past decade, and this has affected how end-of-life care is perceived and delivered [1, 2]. For intensive care unit (ICU) nurses, end-of-life care can be challenging because their role often suddenly shifts from providing life-sustaining care to end-of-life care, specifically through withholding or withdrawal of life-sustaining treatments (WLT) [3, 4]. In the absence of specific training and systemic protocols in place at their hospital for carrying out WLT orders and end-of-life care, ICU nurses face stress-producing uncertainty, ambiguity, and even moral distress about their identity and role as healthcare professionals [5, 6].

In South Korea, the Life-Sustaining Medical Determination Act includes hospice and palliative care so that patients can continue to receive such care even if they decide to withhold or withdraw life-sustaining treatment (LST) [7]. Although the Life-Sustaining Medical Determination Act came into effect in South Korea in 2018, much criticism persists about the way the law came into being, specifically that the legislation was enacted without serious social discourse and effort to reach a consensus on the interpretation of human dignity at the end of life, self-determination, the duty of a nation to protect life, and the right to life [8, 9]. The Act does not even distinguish between withholding and withdrawal of LST, unlike other countries’ Acts [7]. The Act has fundamental limitations regarding ensuring a dignified death because it simply focuses on whether to decide on WLT, ignoring other factors such as dying a dignified death in a quiet, peaceful environment with close family and friends [10]. Therefore, essential questions remain: Was ethical justification for WLT really obtained? and Should other factors not be considered to ensure a dignified death?

Furthermore, in some critical care settings where the discussion of death determines whether a patient receives active treatment, shared decision-making among the patient, family, and healthcare professionals rarely occurs and often without an in-depth and holistic understanding of dignified death [10, 11]. For this reason, ICU nurses report that they rarely provide dignified care for dying patients, leading them to confront ethical difficulties, like ethical uncertainty, ethical conflicts, or moral distress, causing them to burnout [5] or even to consider leaving their jobs [12, 13]. Moreover, they report that they experience role confusion and emotional distress [14]. Nurses’ confusion over their roles in end-of-life care is amplified by the ambiguity of the one legal means to which they can refer, the Life-sustaining Medical Determination Act, which does not clearly delineate their role in end-of-life care [2]. Indeed, many WLT decision-making in ICUs focuses only on whether LST is available and whether legal requirements are met [1, 4]. Thus, ICU nurses face a dilemma: They need to care for patients who do not receive active LST, but they also need to care for patients who need active treatment as mandated by law. On top of this, systemic or structural support to help ICU nurses resolve difficulties is lacking, which has been criticised [1, 15]. If unresolved, this lack of support constitutes one of the external factors that threatens the delivery of good care and the development of nursing professionalism [2, 16].

Furthermore, in South Korea, recently, social values changes caused by the wave of Western individualism are reflected in the stark contrast of the number of deaths occurring at home versus those occurring at medical facilities. Traditionally, most elderly people in South Korea died at home because that was where their children took care of them [2, 8]. In 2021, only 16.5% of all deaths in South Korea occurred at home, while 74.8% occurred at medical institutions [17]. It is in this kind of social context that WLT has emerged as one of the most prominent social and ethical issues in healthcare settings. To resolve them, patients, families and healthcare professionals discuss and decide together on treatment and care plans for their loved ones. However, numerous Korean ICU nurses participate little, or not at all, in advanced care planning [2, 18]. Their roles in end-of-life care are also limited, including WLT decisions [4, 19]. While striving to provide dignified end-of-life care for patients who have decided on WLT, Korean nurses have reported that they experience ethical challenges due to various external factors like poor communication among stakeholders, lack of structural or organisational support, and even their own lack of competency in end-of-life care [2, 4].

Thus, the goal of our research was to analyse ICU nurses’ discourses by addressing three questions: (1) How do ICU nurses view their roles and identities when caring for patients undergoing WLT, and what language do they use to express their views? (2) What ethical issues do ICU nurses face when caring for patients undergoing WLT? (3) How do these discourses enable or limit their professional roles as caregivers for patients undergoing WLT?

## Methods

We used discourse analysis, which takes spoken or written language as data and then analyses how it is used to reach social and cultural perspectives and identities [20, 21]. It comprises a set of tools to analyse participants’ language-in-use to explore broader social and historical contexts of meaning [21, 22], in our case, how nurses’ personally, socially, and professionally derived discourses define their roles as healthcare agents in end-of-life situations. Analysis of in-depth interviews identified discourses that defined ICU nurses’ position in caring for patients who undergo WLT. This analysis was done to determine how patients’ death is handled within the social and ICU system and how ICU nurses who carry out their care are positioned at the frontline of end-of-life care. Quotations of the interviewed nurses are provided to exemplify the types of language-in-use and categories of discourse.

### Participants and settings

Purposive sampling was used to recruit ICU nurses (*n* = 20) who could provide the most insightful information on the issues we were studying [23]. Participants worked in three university hospitals in Seoul and Gyeonggi province in South Korea that had at least 10 ICU beds. All 20 participants who were interviewed had knowledge and experience in caring for patients undergoing WLT in their ICUs.

### Data collection

Data were collected through semi-structured interviews that were based on the framework and specific questions developed by Gee in his discourse analysis toolkit [20, 21] (Appendix 1). Each participant was interviewed one or two times for a total of 60 to 90 min for each interview between December 2021 and February 2022. All participants voluntarily agreed to a face-to-face interview and to have their interview recorded. Considering that the COVID-19 pandemic largely coincided with data collection, interviews were conducted in a consulting room with appropriate virus-transmission preventive measures.

### Data analysis

There is no general consensus on precisely how discourse analysis should proceed [20]. However, Schneider’s ten work steps [24] have been applied successfully to generate new findings about nurses’ constructions of spirituality and how they relate to their clinical practice. Schneider’s ten work steps systematically and efficiently analyse interview texts in the spirit of the work of Norman Fairclough, who is a founder of discourse analysis. To develop components of successful discourse analysis, Gee [20] suggested posing six questions about seven topics; he suggested 28 tools for discourse analysis. Thus, we used Schneider’s ten work steps [24] and Gee’s suggestion on how to carry out a successful discourse analysis [20]. Gee’s Tools of Inquiry [21] were used as an identity-building tool. The analysis process is presented in Appendix 2.

The six questions were composed according to the following domains or themes of Gee [21]: (1) situated meaning; (2) social language; (3) figured world; (4) intertextuality; (5) the big ‘D’, Discourse; and (6) the big ‘C’, Conversation. The definitions of these six domains are summarised in Appendix 1. “Figured world” refers to Holland et al.’s [25] concept about what constitutes and affects identity. Social interactions form figured worlds, and people ‘‘figure out’’ their identity in relation to those around them.

For the identity-building tool, we constructed an analysis framework by referring to Gee [21], which allowed us to explore how one constructs identities and activities with others and how one perceives the identities and activities built around one [20]. The specific questions are presented in Appendix 1.

The interviews were transcribed verbatim and reviewed by the researchers for accuracy against the original recordings. The interviews were independently analysed by two researchers (S.O. and Y.O.). There is a thematic element to data analysis in which the actual language-in-use was interrogated using tools that set up what was being constructed in, by, and through language. The coding process of the texts focused on participants’ language-in-use, with particular attention being paid to repetitive and prominent phrases and expressions that captured the identity of ICU nurses when caring for patients undergoing WLT. These constitute the Discourses’ data units. We repeatedly referred to Gee’s analysis framework [21] to maintain consistency in the analysis. The data units of prominent phrases and expressions were identified and interpreted according to the context. We repeatedly confirmed the relationship between data units and Gee’s framework. The data analysis proceeded via periodic meetings with the other researchers; at these sessions, we continually “moved” back and forth between data sources, coding, and analysis to avoid becoming fixated on an initial, provisional discourse identity. Selected language-in-use phrases were translated into English for the purpose of this paper.

### Rigour

Like other qualitative approaches, discourse analysis involves an interpretive process that can produce multiple interpretations of a given text [20]. Therefore, to enhance the rigour of our study, we subjected our interpretations to key questions suggested by Crowe [26]. To enhance the study’s methodological rigour, we considered how death is treated in our contemporary society. Thus, in the context of the medical community’s legally stipulated way of dealing with death, it is acknowledged that nurses are at the forefront of caring for patients in the ICU, and here, death is often one of the most prominent issues faced by healthcare professionals. Following the discourse analysis stages of Schneider [24], we collected data and analysed it according to research questions suitable for discourse analysis. The social, political, and clinical positions related to these research questions were analysed. Then, the framework developed by Gee [20] enabled systematic analysis, as it provides structure and guidance to determine how one’s “reality” is constructed through discourse.

To support the rigour of our interpretations, we provide verbal texts that adequately explain the relationship between discourse and interpretation; these texts also support the results of the findings. In addition, we made an effort to interpret and explain the relationship between our research findings and previously acquired knowledge about how ICU nurses view death and how they deal with death and dying in the context of providing good nursing care.

## Results

The characteristics of the participants are presented in Table 1. The discourse analysis of participants’ interviews revealed four main kinds of discourses related to (**1**) both “left hanging or feeling abandoned ICU nurses and patients undergoing WLT; (**2**) socially underdeveloped conversations about death and dying management; (**3**) attitudes of legal guardians and physicians toward the dying process of patients undergoing WLT; and (**4**) provision of end-of-life care according to individual nurses’ beliefs in their nursing values (Appendix 3). These will now be considered in turn.

**Table 1:** Baseline characteristics (*n* = 20)

Categories	n (%) or M ± SD
Age (year)	30.1 ± 3.06
Gender	
Male Female	7 (35) 13 (65)
Departments of intensive care unit	
Medical Surgical Emergency Cardiac Neurological	6 (30) 1 (5) 9 (45) 1 (5) 3 (15)
Education level (%)	
Graduate school Bachelor degree Associate degree	1 (5) 17 (85) 2 (10)
Years of nursing experience	4.68 ± 3.31

(M ± SD: Mean ± Standard Deviation)

### Both ‘left hanging’ or abandoned ICU nurses and patients undergoing WLT

The nurses stated that for patients undergoing WLT, physicians became indifferent towards the patient and the nurses once WLT began. Most physicians no longer prescribed medication related to medical treatment for patients undergoing WLT; they were unmotivated and unresponsive to nurses’ demands to check their patients who needed physicians’ examinations or treatments. Physicians appeared to be well aware that even if they act like that, there are no legal or administrative restrictions on their behaviour. This situation made ICU nurses feel helpless and abandoned because their efforts to provide appropriate medical treatments were hindered by the physicians’ indifferences; for example, they often did not respond to nurses’ calls or prescribe appropriate medical examinations or treatments. Indeed, most physicians were unresponsive when nurses alerted them that the patients were suffering while undergoing WLT. Some physicians would not even try to improve end-of-life care for patients undergoing WLT.

Analysis of nurses’ language-in-use indicates that ICU nurses were confused. Examples include phrases such as “a similar case but a different decision depending on the doctor in charge of the patient”; “I hope doctors to carefully check my patients undergoing WLT and give a needed treatment to relieve my patients’ sufferings”; and “what would be right to do as a nurse in such limited situations”. It created a figured world that threatened ICU nurses’ professional identity; they were regarded as detached persons who merely watched the patients’ sufferings caused by a lack of adequate medical treatment from the sidelines, offering little to the discourse on WLT decisions or feeling hamstrung to provide any meaningful care. In this regard, nurses felt that their professional identity was also threatened. For example:

When a patient’s vital signs are highly unstable, we did actively alert the doctor before, but now? I do not actively notify doctors because of their “no responses.” Nowadays, in that case, I provide nursing care as best as I can to solve such patients’ problems. But, nursing care sometimes would not be the solution to fundamentally improve patients’ medical conditions. That is, there are always limitations unless doctors prescribe. As a nurse, when I watch patients [undergoing WLT] suffering from a lack of proper medical treatment, I realise that no matter how hard I try, nursing alone can’t alleviate their suffering. It’s tough. I don’t know the right thing to do for dignified care in caring for the patients [undergoing WLT].

Nurses are qualified to provide dignified end-of-life care. However, they still lack the authority to get involved in treating patients undergoing WLT. The physician determined the feasibility of resuscitation, the legal guardian gave up certain rights, and they both decided on when to initiate WLT. The ICU nurse, on the other hand, was left on the “sidelines”, observing others’ decisions, wanting to help but not allowed to. For example:

As a nurse, I don’t feel like I have to do anything more for this patient undergoing WLT in the ICU, but how can I just wait until the patient’s heart rate drops to zero BPM while the patient is still there? What I do is let the legal guardian visit the patient […] At that time, I wondered if I could really do this.

Another language-in-use phrase used in these situations was “nursing efficiency and low priority regarding care for patients undergoing WLT.” This discourse was pervasive in the data. Language-in-use phrases associated with this notion, a management system focusing on the maximisation of treatment efficiency and no laws regarding end-of-life care for patients undergoing WLT included: “organisational climate, managers, and physicians forced me to focus on the other ICU patients rather than to provide end-of-life care for patients undergoing WLT”; “more efficient to provide nursing care to other patients who can possibly recover”; and “no regulations or guidelines about end-of-life care for patients undergoing WLT, no system or physicians seem to care about their dignified death”. These phrases build a world within the ICU system that is at odds with helping patients die in a dignified manner. In addition, it does not align well with the basic ethical right of respecting human dignity since the nurses argued that all human beings have the fundamental right to receive the best nursing care, regardless of their health status. Furthermore, the ICU is always overflowing with patients in need of intensive care. As a result, nurses did not give priority care to patients undergoing WLT. For example:

Considering our ICU atmosphere, the unit manager and doctors want me to focus more on other, more critically ill patients. Patients undergoing WLT should receive good end-of-life care, but here, I am forced to care for the other ICU patients, I mean, patients not undergoing WLT, that is, patients who are hopeful of recovering. I am frustrated, and it is confusing and difficult at first, but now I also care for other patients who need intensive care, not patients undergoing WLT.

In summary, physicians’ indifference towards dying patients after the initiation of WLT was associated with ICU nurses’ language-in-use phrases describing role confusion. Another language-in-use phrase commonly used in this context was “lack of authority, organisational climate, and low priority regarding care for patients undergoing WLT”, which meant current nursing care for WLT patients in the ICU was in conflict with their nursing values of protecting human dignity. Ultimately, it threatened their professional identity.

### Socially underdeveloped conversations about death and dying management

ICU nurses often wondered whether the patient undergoing WLT really knew what WLT meant or how they might suffer at the end of life. ICU nurses witnessed that patients and their legal guardians did not readily talk about death prior to making the decision to initiate WLT. The nurses also observed that healthcare professionals had few or no discussions about patients’ deaths. ICU nurses highlighted that, in South Korea, discussing death tends to be taboo. This stance is related to a superstition that such specific discussions might actually facilitate death. It can also be viewed as an adult child’s mistreatment of their parents. Nurses’ conversations about their patients’ death were pervasive throughout our discourse data. That meant that during their lifetime, patients might not have discussed or pondered about their death sufficiently or might not have discussed their death with family members. Similarly, patients and their families might not have sufficiently considered the meaning and consequences of WLT. For example:

The legal guardians have already told the medical staff that the patient’s condition is not very good. There may be financial or psychological issues if the period [length of WLT care] is already too long or if this [or that] happens. They [legal guardians] are physically and psychologically very tired. And in fact, there is no hope. But, most of the patients’ legal guardians seem not to talk with them [the patients] about WLT.

ICU nurses frequently observed the suffering of patients undergoing WLT who ended up not dying soon after WLT was initiated. For ICU nurses, providing medical treatment under these circumstances could be considered more ethically problematic if their treatment conflicts with the patient’s wishes for WLT.

Under these circumstances, the nurses’ language-in-use reflected a sense of ethical confusion: “Should we provide suction lung care to a sleeping patient?”; “I don’t know what the patient is thinking, but I may not want it if I were that patient“; “Who is this treatment for [the patient or legal guardian]?”; and “Did the patient know the meaning of their decision to withdraw life-sustaining treatment and what it means to sign the legal document?” This ethical ambiguity created a figured world that constantly challenged the professional identity of ICU nurses. Within this ICU environment, nurses are viewed as individuals who endure constant questioning about their treatment to justify the care of their patients undergoing WLT. ICU nurses were confused and conflicted as they tried to determine the direction of the end-of-life care they would provide patients undergoing WLT. It was especially trying for them ethically, as they had to witness the patients’ end of life and eventual death without having time to interact with those patients for end-of-life care.

ICU nurses were sceptical about temporarily prolonging a patient’s life by maintaining mechanical ventilation. Simultaneously, however, they wondered whether prolonging a patient’s life, even for just a few minutes, would really be in the best interest of the patient. This dilemma is exemplified by the following quote:

But in another way, it just seems meaningless. After all, it [the treatment] may not be for the sake of the patient. On the other hand, I sometimes get a little bit confused as the patients might desire to prolong their lives. Because of a lack of discussion or deliberation among them [patients and the legal guardians]. I used to wonder whether they had enough time to talk about it.

In summary, traditional Korean taboo conversations about death and dying were associated with ICU nurses’ language-in-use phrases describing confusion and conflict on how to proceed with carrying out WLT procedures and compatible WLT care.

### Attitudes of legal guardians and physicians toward the dying process of patients undergoing WLT

The ICU nurses’ language-in-use showed that legal guardians and physicians were not yet prepared to deal with dying patients undergoing WLT, and their attitudes were indifferent. For legal guardians, physicians, and nurses, the decision to initiate WLT represents a critical turning point affecting their relationships with patients, and the effect was abrupt. Furthermore, they scarcely discussed how to deal with the patient’s dying process or who might decide on WLT unless the patients were incapable of deciding for themselves.

#### Legal guardians’ lack of knowledge and attitudes toward the dying process of patients undergoing WLT

The language-in-use indicates that legal guardians lacked knowledge or had indifferent attitudes toward dying patients undergoing WLT despite consent for WLT. For instance, “legal guardians commonly ask nurses why patients aren’t dead yet” or “I know that WLT is the same as physician-assisted suicide or euthanasia”. This caused nurses to feel frustrated and compassionate toward their patients. The following is an example of a legal guardian’s attitude toward the treatment of patients undergoing WLT.

Most of the legal guardians think that death will come soon after they complete filling out the documents that terminate life-sustaining treatment. After the patient’s decision was made, when the legal guardian told me to stop giving oxygen to the patient, to not feed them, to not do anything, I had a really hard time. I explained to the legal guardian that this was not the real meaning of WLT, to also withhold basic oxygen and nutritional requirements. Whenever I see such, [I] feel too bitter. [I] feel sorry for these patients every time I see them.

#### Physicians’ hesitancy to make WLT decisions and restrict palliative care

The language-in-use analysis showed that physicians were hesitant or sometimes reluctant to make WLT decisions. Language in this category of discourse was exemplified by phrases such as: “doctors hesitate to sign legal documents”; “doctors were passive in signing the legal document”; and “It’s really heartbreaking”. The nurses understood the physicians’ attitudes as they witnessed legal difficulties due to unspecified and ambiguous laws. Nevertheless, they highlighted that, ultimately, physicians’ attitudes had an impact on caring for dying patients undergoing WLT, which caused nurses to experience uncomfortable feelings. This discomfort is exemplified by the following excerpts:

If doctors were legally protected against failing to actively treat them [WLT patients], and [if] doctors didn’t get sued for that failure, they would have followed their hearts and signed [the form for WLT]. But doctors have experienced legal difficulties or have witnessed legal difficulties of other doctors [in the past], so maybe they are reluctant to sign documents. Even so, such attitudes impact caring for patients undergoing WLT; I mean, due to them, nursing care is very restricted. When lacking palliative care, I am the only person who witnesses dying patients’ suffering. It’s really heartbreaking, but [there is] not much I can do for my patients, so I feel sorry for them.

In summary, the indifferent attitudes of legal guardians and physicians toward the dying process of patients undergoing WLT were associated with ICU nurses’ language-in-use phrases describing psychological discomfort.

### Provision of end-of-life care according to individual nurses’ beliefs in their nursing values

Legal documents for WLT explain end-of-life care in insufficient detail and are limited in describing the quality of care. Therefore, nursing care should be entirely entrusted to nurses’ ethical values or responsibility. Each nurse strives to provide dignified end-of-life care according to their personal beliefs about nursing values.

Language-in-use emphasises that palliative care guidelines for patients undergoing WLT are unclear. However, the nurses provided end-of-life care based on their beliefs in nursing values: “if the patient is conscious”; “only if the patient is in [a] coma”; “if the doctor doesn’t set a scope of treatment, I’ll do it myself and do the best I can”; and “the right to receive dignified end-of-life care from their nurses”. Nurses’ approach to caring for patients undergoing WLT are depicted in the following excerpts:

If the doctor doesn’t set a scope of palliative care or is reluctant to get involved in it, I’ll do it myself and do my best to alleviate my patients’ pain and comfort them. As a nurse, I feel compassion toward them [their patients with WLT] and deliver end-of-life care for my patients because they have the right to receive dignified end-of-life care from their nurses.

What kind of care routines do ICU nurses carry out with patients undergoing WLT? The answer varies for each nurse, as the nurses did not find justifiable grounds for their routine nursing practices. For physicians, their role in caring for these patients is more clear-cut. By law, they are prohibited from resuscitating patients with WLT directives. Therefore, physicians are more passively involved in maintaining the lives of patients undergoing WLT. For nurses, their role is less clear-cut, as there are no definitive guidelines to direct their care of patients undergoing WLT. As such, nurses experience ambiguity about how much they should be involved in the physician-patient care relationship. This ambiguity is reflected in the following quotation:

It would be helpful if there were clear guidelines on how to care for patients undergoing WLT in the ICU. But, we have unclear and lack of authority regarding end-of-life care for my patients by the Act. But, it’s contrary to nursing values. So, that’s why I just provide end-of-life care for my patients, following what I’ve learned and what I believe about nursing. But, I still get confused and doubt whether my actions are right or not by the law.

End-of-life care for patients undergoing WLT was based on the best nursing-care model, which took into account the personal convictions and training of individual nurses. Although ICU nurses strived to provide the best possible care, they often failed to provide this as they were obligated to care for other patients who were in greater need, causing feelings of guilt. Nonetheless, on a case-by-case basis, nurses constructed individual care frameworks based on their personal experience, knowledge, and beliefs about nursing care so that they could minimise their guilt:

I often have to care for other patients who need my help, so I feel bad every time I don’t provide good end-of-life care for my patients, but I seek to find solutions. It’s a kind of self-defence mechanism. It’s my one and only solution to keep me going. But anyway, one of my routines is to watch the elevator doors until closing when my patient goes to the mortuary. It is my own way of giving last end-of-life care and respecting [the patient’s] dignity for the very last time.

In summary, the lack of roles for nurses specified in the law for WLT and the indifference of physicians regarding patients undergoing WLT were associated with ICU nurses’ language-in-use phrases associated with the provision of end-of-life care according to individual nurses’ beliefs in nursing values.

## Discussion

Our present study examined how discourses of ICU nurses in South Korea shape their roles as nurses tasked with caring for patients who request and undergo WLT, specifically how they mould and enable or limit them in their professional duties and identity. The main context in which these discourses occurred is societal and structural problematic: little-discussed issues of death and dying in Korean society and in the ICU hospital system, and ambiguities of the 2018 Life-Sustaining Medical Determination Act of South Korea and how it should be applied. Through the use of discourse analysis of texts from semi-structured interviews of ICU nurses, we identified four themes of discourse: (**1**) both “left hanging or feeling abandoned ICU nurses and patients undergoing WLT; (**2**) socially underdeveloped conversations about death and dying management; (**3**) attitudes of legal guardians and physicians toward the dying process of patients undergoing WLT; and (**4**) provision of end-of-life care according to individual nurses’ beliefs in their nursing values.

Our participant nurses’ language-in-use reflects the ambiguity of their roles in the ICU as a threat to nursing values, one that is obscured by the ever-changing medical approaches to dealing with the deaths of patients undergoing WLT. Furthermore, “unwritten” regulations and the vague Act tend to persuade ICU nurses to limit end-of-life care. This is to focus hospitals’ care management system based on the maximisation of treatment efficiency and physicians’ stance on critically ill patients not undergoing WLT and patients who have the hope of recovering. Our findings are consistent with other studies investigating difficulties in providing end-of-life care among ICU nurses in other countries [27, 28]. Nasu et al.’s systematic review of end-of-life care practice also highlighted the need for proper legal documentation regarding nurses’ roles and authority in dignified end-of-life care [29]. This review found that nursing care responds to the multiple demands of patients, families, and physicians, and nurses have many responsibilities for evaluating and addressing patients’ medical concerns; however, they lack the authority to deal with end-of-life care regardless of country or cultural contexts [29]. Baykara et al. [30] argued that the primary reason for difficulties experienced by Turkish ICU nurses regarding end-of-life decisions was a lack of specific legal regulations. Valiee et al. [31] reported that Iranian nurses’ involvement in end-of-life and ethical decision-making processes was well below the expected levels due to the absence of legal regulations. DuBois and Reed [32] stressed that changes in policy are needed to enable United States nurse practitioners to reach their full scope of practice in a way that benefits patients and families at the end of life. Health policymakers should consider amendments to the laws that restrict end-of-life nursing care for patients undergoing WLT and establish regulations to protect and promote the rights of patients undergoing WLT to receive dignified end-of-life nursing care.

Furthermore, the ICU climate that focused on maximising treatment efficiency pressured the nurses to care for other patients who required intensive care. Such a context created a figured world which caused confusion about their roles based on nursing values of protecting human beings’ dignity. Ultimately, this threatened their professional identity. This aligns with previous findings regarding nurses’ experiences with end-of-life care [5, 33, 34]. Kim et al. [5] explored ethical conflicts among nurses in geriatric hospitals and found that they felt disillusioned with hospitals’ attitudes that considered treatment as only for financial benefit. Some left their positions or quit their jobs because they realised their limited power and were unable to change their organisations’ systems or stances. Oh and Gastmans’s systematic review on ethical issues among ICU nurses caring for patients with COVID-19 reported that the most distressing issue in end-of-life care involved threatening patients’ dignity [14]. This consistency can be attributed to the moral distress experienced by nurses. Moral distress refers to the inability to do the right thing despite being aware of it [35]. Organisational climate conflict with nurses’ beliefs in nursing values causes moral distress and damages moral integration, a component of professional identity [36, 37]. ICU nurse leaders should establish a climate that supports nurses’ beliefs about nursing values. It is crucial to listen to them so that they can do the right thing and advocate as moral agents by providing dignified end-of-life care for patients undergoing WLT.

Our participants felt frustrated in situations where they perceived that physicians’ indifferent attitude toward end-of-life care hindered providing dignified nursing care for their patients. Our findings are supported by previous studies of nurses’ moral distress involving physician practice [36, 38]. Indeed, care approaches through poor communication between physicians and nurses contradict patients’ wishes, and nurses are restricted from participating in the determination and planning of end-of-life care for their patients [39, 40]. In such situations, nurses experience moral distress because of the power structure of the healthcare setting. Furthermore, the Korean nursing environment is dominated by medical paternalism and hierarchism, which restrict nurses’ authority in caring for patients. In cultural contexts or hierarchical power structures similar to those of hospitals in South Korea, medical paternalism results in moral distress among nurses in Taiwan [41], Iran [42], and China [43]. According to Prompahakul and Epstein’s integrative review of moral distress among non-Western nurses [44], although both Western and non-Western healthcare settings have professional hierarchical structures, the extent might differ in non-Western contexts. Kovanci and Akyar [34] analysed the influence of cultural context on moral distress among ICU nurses and concluded that moral distress is similar in countries with similar cultures and those which are geographically close. Given such culturally distinctive traits, further discourse analysis research reflecting cultural contexts in exploring moral distress among ICU nurses who care for patients undergoing WLT is required.

An analysis of language-in-use reveals conversations (Big “C”) [20] regarding a lack of social discourse about caring for patients undergoing WLT in South Korea. It also takes place within the ICU setting where care for WLT patients is provided. Patients, legal guardians, and even healthcare professionals are not accustomed to discussing the meaning or processes of WLT and the necessary palliative care before deciding on WLT. Due to these social contexts, nurses experienced confusion about their roles and doubted whether their care would be in the best interests of their patients. Their language-in-use can be explained as moral uncertainty, which “arises when one is unsure what moral principles or values apply, or even what the moral problem is” [45]. Moral uncertainty occurs when ICU nurses are obliged to work despite unclear guidelines or regulations regarding the treatment of their patients [30, 31]. Indeed, during the COVID-19 pandemic, numerous nurses experienced moral uncertainty as they queried what principles should be applied to resolve ethical issues [14]. This was because of inconsistent healthcare policies or the absence of guidelines for dealing with ethical issues such as protecting human rights [14]. The establishment of clear guidelines or rules can alleviate confusion among nurses due to moral uncertainty, thereby promoting their professional commitment to nursing care [46] and quality of care [47].

Although ICU nurses were confronted with obscure laws and guidelines and a lack of authority for end-of-life care for patients undergoing WLT, each nurse strived to deliver good nursing care by following their nursing values and protecting human dignity. This enabled them to maintain their professional identities and avoid guilt. Remarkably, they described themselves as the only ones who could be concerned about and represent their patients’ human rights and dignity. They made an effort to fulfil the role of advocates for their patients and were able to do so because they were beside their patients and vividly witnessed their suffering. Our findings align with those of other studies on ICU nurses’ ethical behaviour based on nursing values, despite clashes with the organisational climate or physicians’ and legal guardians’ interests [39, 48]. Flannery et al. [39] found that Australian ICU nurses tended to advocate on behalf of the patient and what they interpreted as the patient’s best interest. Peter et al. [48] revealed that Canadian ICU nurses endeavoured to fulfil their caring responsibilities as moral agents through face-to-face relationships. Such consistency may originate from a strong sense of ethical and professional responsibility among nurses. Norvedt [49] asserted that epistemological knowledge and ontological essence in nursing are fundamentally involved in ‘being-for-the-other’, which is being responsible for another by responding to their suffering and vulnerability, namely, ethics. Nurses’ responsibilities originate from the patient’s appeal and arise from a response to the patient’s moral reality [49]. Martinsen [50] emphasised that nurses feel an ethical responsibility arising from patients’ demands through ethical meetings. However, according to a recent review of ethical responsibility among nurses, few empirical studies have investigated it [51]. Further studies are needed to explore the essence of nurses’ responsibility for developing competence in ethical and professional responsibilities.

### Implications

Using discourse analysis, this research highlights the difficulties and ethical challenges nurses experience in providing care to patients undergoing WLT. The findings can provide us with a key to recognising the needs of social discourse and the amendment of related laws in caring for patients undergoing WLT. Thus, our findings indicate that it is vital for healthcare policymakers and nurse leaders to listen to nurses’ voices so that they can provide dignified end-of-life care for patients undergoing WLT as moral agents. Our suggestions can inspire approaches or ideas to reduce moral uncertainty and alleviate moral distress among nurses, which can advance the development of their professional roles and identities, ultimately contributing to the protection and promotion of dignified dying and death of patients undergoing WLT.

### Limitations

We acknowledge the limitations in generalising the findings of our small-scale study. However, our research offers new insights into a previously overlooked aspect in the field of how nurses view their professional roles and identities in end-of-life care for patients undergoing WLT. The frontline accounts of 20 nurses who volunteered to participate in our study likely do not encompass all the possible perspectives, nor do we believe that our interpretations of the participants’ language-in-use are the only valid interpretations. Furthermore, there could be differences in nurses’ experiences of caring for patients undergoing WLT depending on cultural or regional contexts. In addition, nurses’ interviews might not have been fully reflected due to translation limitations of their interviews from Korean to English. Instead of providing absolute certainty, readers are encouraged to assess the significance of our findings by considering how they align with their own experiences and our explanation of the research methodology.

## Conclusions

This study examined how the discourse of ICU nurses in South Korea shapes their role in caring for patients undergoing WLT, specifically, how they enable or limit their professional roles and identity. We hope that the discourses provide another perspective on the challenges faced by ICU nurses caring for patients undergoing WLT. For example, a lack of social discourse, obscure laws regarding caring for patients undergoing WLT, and societal contexts influence nurses’ attitudes or approaches to caring for patients undergoing WLT in the ICU. In such environments, ICU nurses experience role confusion and psychological distress, such as emotional distress and moral distress, which could affect their professional identity. However, they recognised patients’ vulnerability and felt compassion toward them. Individually, each nurse strived to provide good care as an advocate according to their nursing values. Their strong sense of responsibility enabled them to provide dignified end-of-life care despite situational constraints. For the dignified dying and death of patients undergoing WLT, it is necessary to encourage social discourse on what a dignified death means and how end-of-life care is delivered and to amend the related laws by reflecting the perspectives of stakeholders, including nurses. Achieving them will move us closer to answering ICU nurses’ questions about how we should go about providing end-of-life care for patients undergoing WLT.

### Electronic supplementary material

Below is the link to the electronic supplementary material.

Supplementary Material 1

## Data Availability

Data are available upon request from the corresponding author of the study.
