# Supplementary material for: Care of patients undergoing withdrawal of life-sustaining treatments: an ICU nurse perspective
Source: BMC Nurs. 2024 Mar 4;23:153. doi: 10.1186/s12912-024-01801-7 (PMC10910717; doi:10.1186/s12912-024-01801-7)
Supplement: Supplementary file 1 — Supplementary Material 1 [file 12912_2024_1801_MOESM1_ESM.docx]

| Appendix 1. The framework and specific questions to ask data of Gee for analysis | | |
| --- | --- | --- |
| **Domain** | **Definition**  **(Adapted from Gee, 2014)** | **Questions for building tasks**  **(Gee, 2014; 2014)** |
| 1. Situated meaning | Specific meanings are connected to actual situations where a word or structure is used. | - How do the six domains represent the social relationships of ICU nurses, physicians, and legal guardians in the management of DNR patients in the ICU? - How are the six domains presented to ICU nurses performing the nursing of DNR patients? - How are the six domains used to perform and describe the ICU nurse’s identity in caring for DNR patients? - How are societal influences on the six domains used to build and maintain ICU nurses’ care for DNR patients? |
| 2. Social languages | Varieties or styles of languages connected to enact a particular social identity. |  |
| 3. Intertextuality | Ways of that one text quote, refer to or allude to another text. |  |
| 4. Figured worlds | A picture of a captured world that is accounted to be normal and typical in social and cultural contexts. |  |
| 5. The big ‘D’ Discourses | Communications through time and history by humans enact distinctive social identities. |  |
| 6. The big ‘C’ Conversations | Public debates around social issues around people at specific times and places. |  |

| Appendix 2. The analysis process based on Schneider’s (2013) Work Steps | |
| --- | --- |
| **10 Work Steps** | **Analysis process** |
| 1. Establish the context | Establishing social, cultural, and historical context of DNR care in the ICU. |
| 2. Explore the production process | Investigation of additional information about the background of DNR and ICU, including institutional policy and nurses’ characteristics. |
| 3. Prepare your material for analysis | Transcribing interview recordings and saving them as computer files. |
| 4. Code your material | Coding of scripts manually into specific meaning units of ICU nurses’ identity in DNR care. |
| 5. Examine the structure of the text | Examination of structural features of patterns and relationships among discourse strands. |
| 6. Collect and examine discursive statements | Collect discourse fragments of individual statements in regard to Gee’s Tools of Inquiry: Identity building. |
| 7. Identify cultural references | Identification of intertextuality in the cultural context. |
| 8. Identify linguistic and rhetorical mechanisms | Identification of the discursive function of statements regarding identity building at the level of language, including word, grammar, and rhetorical features. |
| 9. Interpret the data | Interpret the attribute and function of the discourse in the ICU nurses’ identity in DNR care by using Gee’s Tools of Inquiry. |
| 10. Present your findings | Compile results and interpretation and present them in the research article. |

| Appendix 3. Main themes and examples of the quotes | |
| --- | --- |
| **Main themes** | **Quotes** |
| Both “left hanging or feeling abandoned ICU nurses and patients undergoing WLT | *“When a patient's vital signs are highly unstable, we did actively alert the doctor before, but now? I do not actively notify doctors because of their “no responses.”*  *“ I am forced to care for the other ICU patients, I mean, patients not undergoing WLT. I am frustrated, and it is confusing and difficult at first, but now I also care for other patients who need intensive care, not patients undergoing WLT.”* |
| Socially underdeveloped conversations about death and dying management | *“I sometimes get a little bit confused as the patients might desire to prolong their lives. Because of a lack of discussion or deliberation among them [patients and the legal guardians]. I used to wonder whether they had enough time to talk about it.”* |
| Attitudes of legal guardians and physicians toward the dying process of patients undergoing WLT   - Legal guardians’ lack of knowledge and attitudes toward the dying process of patients undergoing WLT - Physicians’ hesitancy to make WLT decisions and restrict palliative care | *“I explained to the legal guardian that this was not the real meaning of WLT, to also withhold basic oxygen and nutritional requirements. Whenever I see such, [I] feel too bitter. [I] feel sorry for these patients every time I see them”*  *“But doctors have experienced legal difficulties or have witnessed legal difficulties of other doctors [in the past], so maybe they are reluctant to sign documents. Even so, such attitudes impact caring for patients undergoing WLT; I mean, due to them, nursing care is very restricted.”* |
| Provision of end-of-life care according to individual nurses’ beliefs in their nursing values | *“We have unclear and lack of authority regarding end-of-life care for my patients by the Act. But, it’s contrary to nursing values. So, that’s why I just provide end-of-life care for my patients, following what I’ve learned and what I believe about nursing. But, I still get confused and doubt whether my actions are right or not by the law.”* |
